# Supplementary figures and images for: Condition-adaptive fused graphical lasso (CFGL): An adaptive procedure for inferring condition-specific gene co-expression network
Source: PLoS Comput Biol. 2018 Sep 21;14(9):e1006436. doi: 10.1371/journal.pcbi.1006436 (PMC6173447; doi:10.1371/journal.pcbi.1006436)

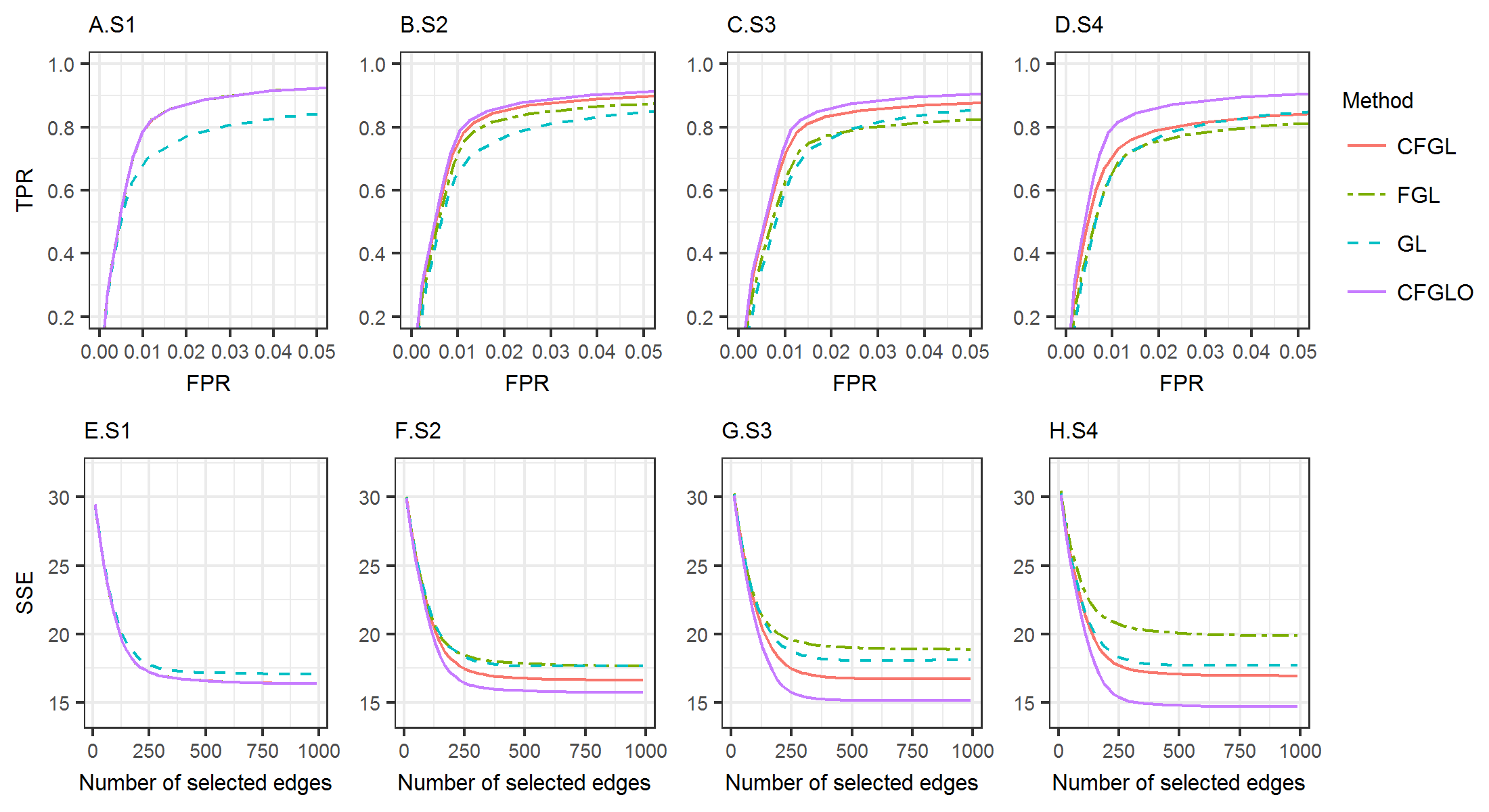

Supplement: S1 Fig — Top row (A-D): ROC curves for edge detection in the four simulation settings (S1-S4). Bottom row (E-H): SSE for edge weight estimation in the four simulation settings (S1-S4). Red line: CFGL, Green line: FGL, Blue line: GL, Purple line: CFGL-oracle. (TIF) [file pcbi.1006436.s001.tif]

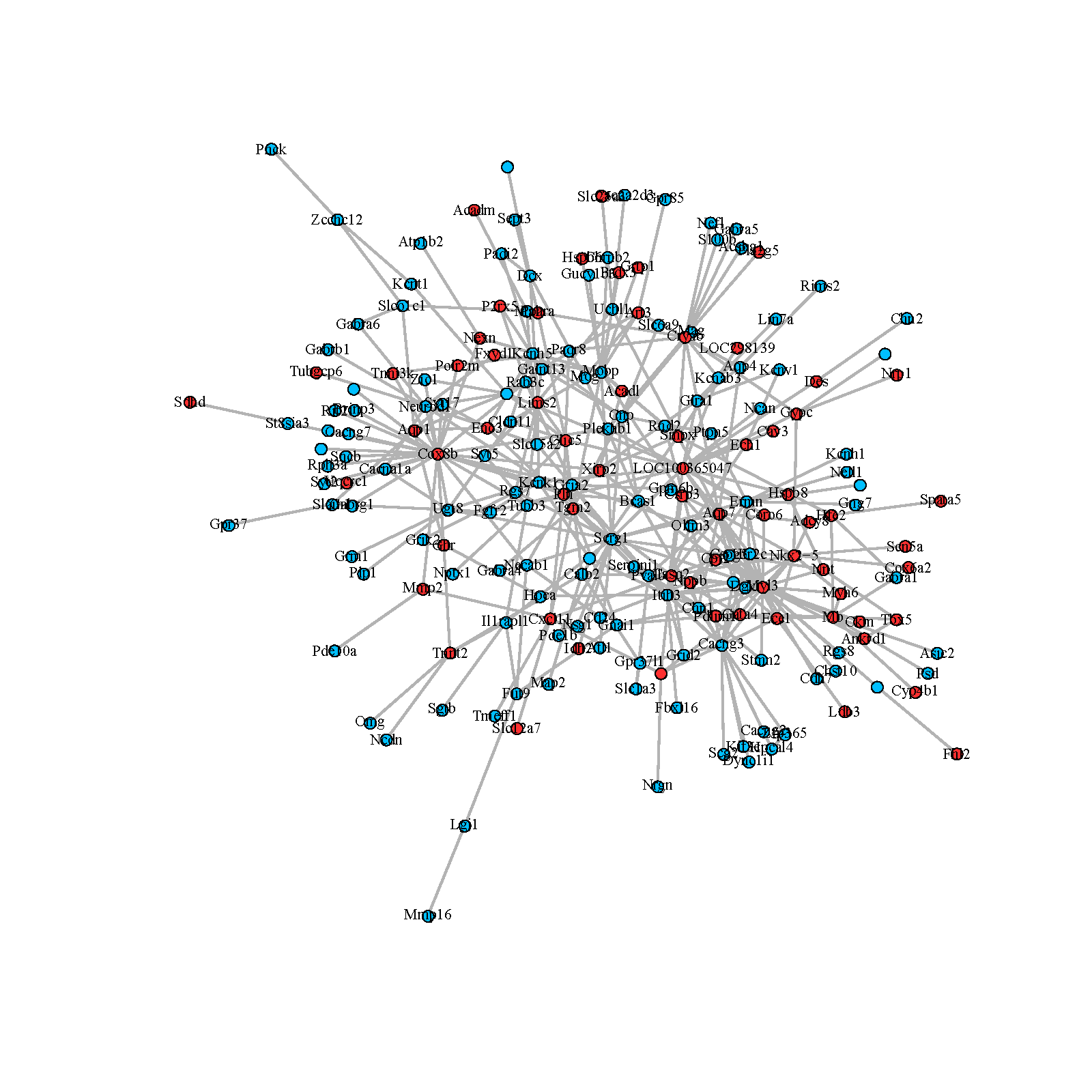

Supplement: S2 Fig — (TIFF) [file pcbi.1006436.s002.tiff]

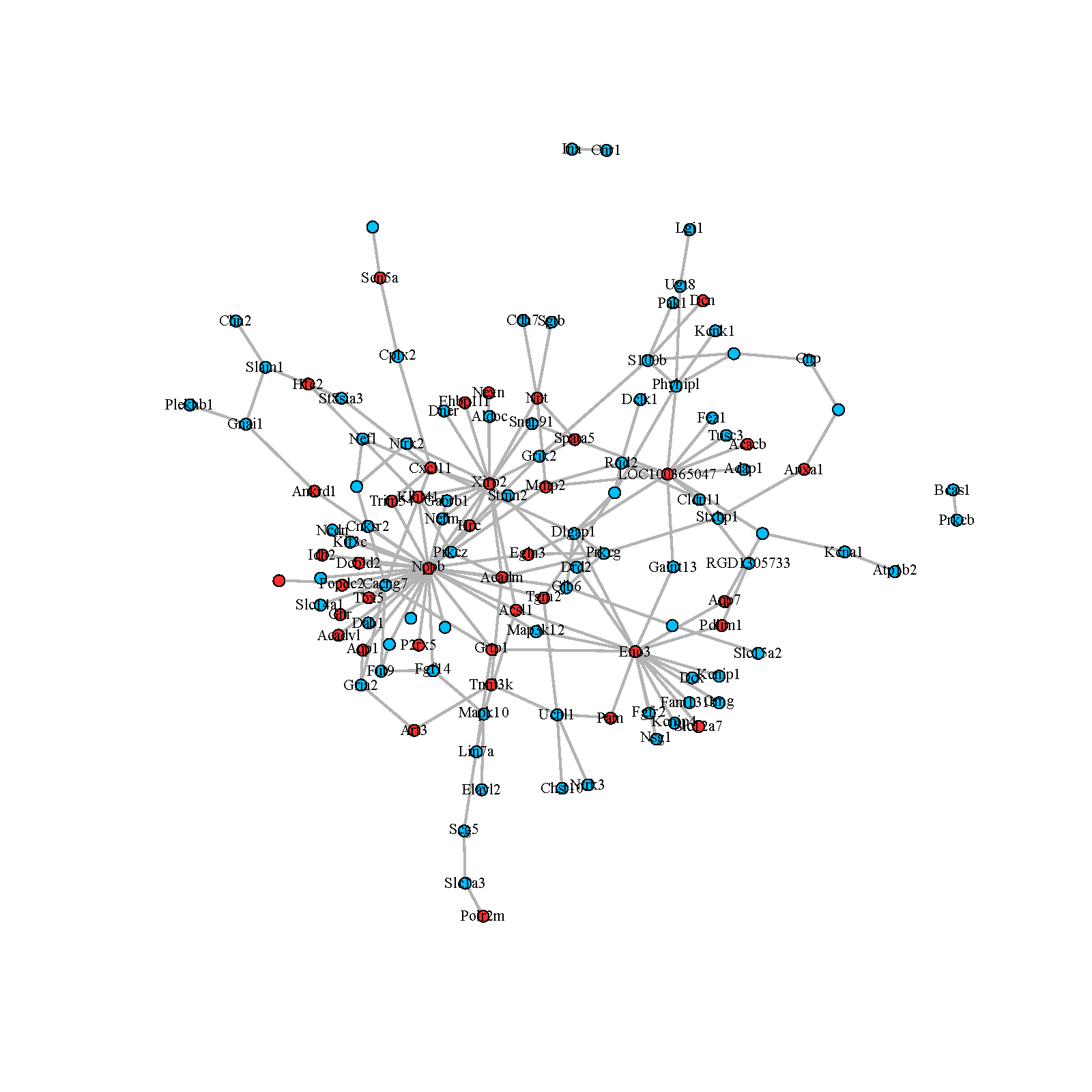

Supplement: S3 Fig — (TIFF) [file pcbi.1006436.s003.tiff]

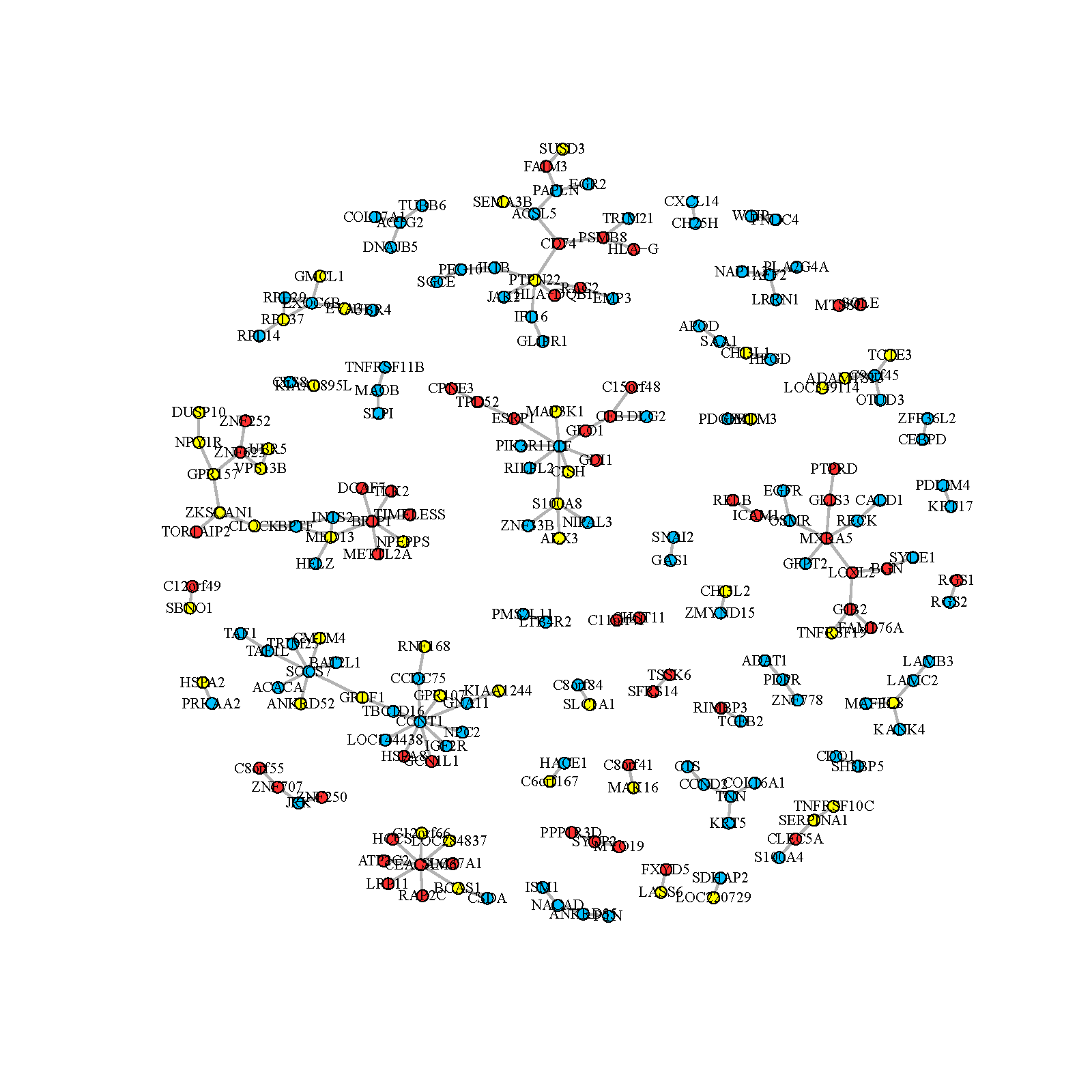

Supplement: S4 Fig — Red: Genes that are up-regulated in both tumor tissues in comparison with normal tissue. Blue: Genes that are down-regulated in both tumor tissues. Yellow: Genes that are up-regulated in one tumor tissue but down-regulated in another. (TIFF) [file pcbi.1006436.s004.tiff]

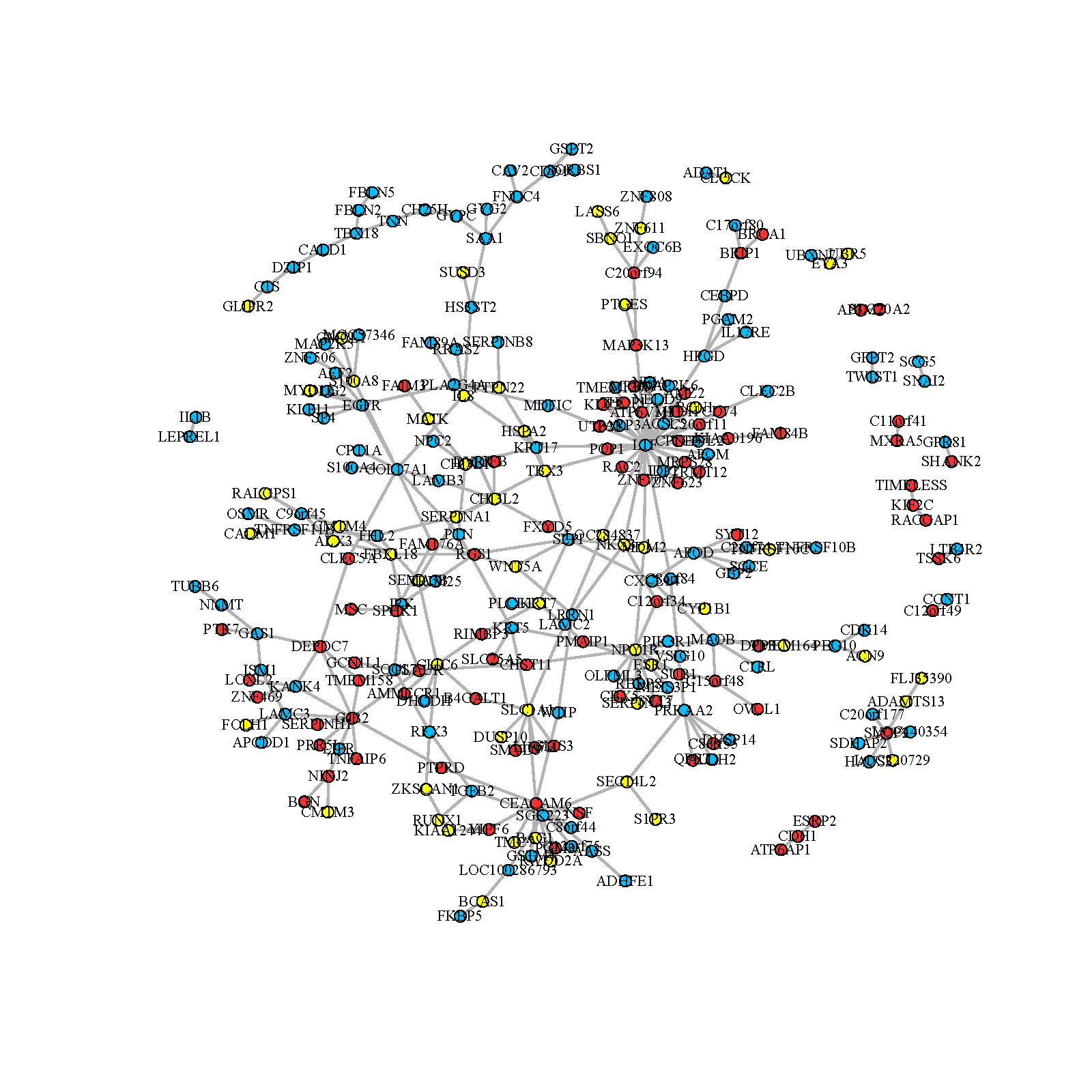

Supplement: S5 Fig — Red: Genes that are up-regulated in both tumor tissues in comparison with normal tissue. Blue: Genes that are down-regulated in both tumor tissues. Yellow: Genes that are up-regulated in one tumor tissue but down-regulated in another. (TIFF) [file pcbi.1006436.s005.tiff]

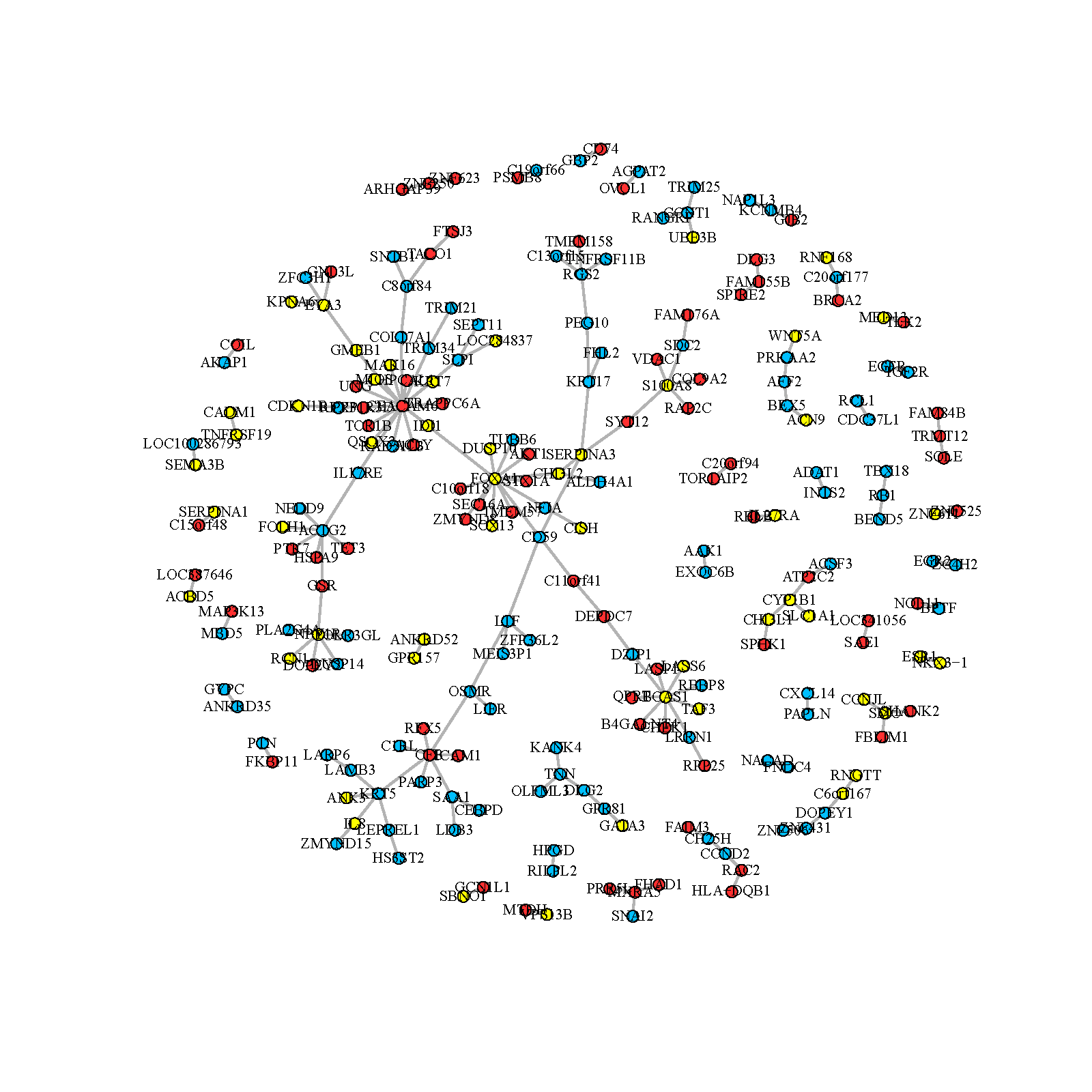

Supplement: S6 Fig — Red: Genes that are up-regulated in both tumor tissues in comparison with normal tissue. Blue: Genes that are down-regulated in both tumor tissues. Yellow: Genes that are up-regulated in one tumor tissue but down-regulated in another. (TIFF) [file pcbi.1006436.s006.tiff]
