# Supplementary material for: Condition-adaptive fused graphical lasso (CFGL): An adaptive procedure for inferring condition-specific gene co-expression network
Source: PLoS Comput Biol. 2018 Sep 21;14(9):e1006436. doi: 10.1371/journal.pcbi.1006436 (PMC6173447; doi:10.1371/journal.pcbi.1006436)
Supplement: S1 Table — (DOCX) [file pcbi.1006436.s007.docx]

**Supplementary Table 1. Comparison of partial AUCs in the 2-condition simulation studies.**

CFGL, CFGLO, FGL and GL were compared under 4 simulation scenarios (S1-S4) with 2 sample sizes (n=50 and 100). The analysis was run on a grid of $\lambda_{1}$ and $\lambda_{2}$. The ROC curves were computed over $\lambda_{1}$ for each fixed $\lambda_{2}$, and the partial AUCs in the FPR range of [0, 0.05] were computed. The table summarizes the partial AUCs for $\lambda_{2}=$0.05, 0.10, 0.15, and 0.20.

| $\lambda_{2}$ | Method | n = 50 | | | | n = 100 | | | |
| --- | --- | --- | --- | --- | --- | --- | --- | --- | --- |
|  |  | S1 | S2 | S3 | S4 | S1 | S2 | S3 | S4 |
| 0.05 | CFGL | 0.650 | 0.641 | 0.644 | 0.619 | 0.786 | 0.748 | 0.765 | 0.740 |
|  | CFGLO | 0.651 | 0.656 | 0.652 | 0.658 | 0.786 | 0.760 | 0.771 | 0.775 |
|  | FGL | 0.651 | 0.619 | 0.634 | 0.606 | 0.786 | 0.719 | 0.752 | 0.721 |
|  | GL | 0.583 | 0.594 | 0.588 | 0.590 | 0.726 | 0.702 | 0.713 | 0.714 |
|  |  |  |  |  |  |  |  |  |  |
| 0.10 | CFGL | 0.692 | 0.663 | 0.676 | 0.626 | 0.811 | 0.762 | 0.784 | 0.739 |
|  | CFGLO | 0.694 | 0.695 | 0.693 | 0.711 | 0.811 | 0.787 | 0.798 | 0.806 |
|  | FGL | 0.694 | 0.621 | 0.656 | 0.600 | 0.811 | 0.704 | 0.756 | 0.700 |
|  | GL | 0.583 | 0.594 | 0.588 | 0.590 | 0.726 | 0.702 | 0.713 | 0.714 |
|  |  |  |  |  |  |  |  |  |  |
| 0.15 | CFGL | 0.711 | 0.671 | 0.688 | 0.620 | 0.817 | 0.764 | 0.788 | 0.730 |
|  | CFGLO | 0.714 | 0.717 | 0.715 | 0.723 | 0.817 | 0.800 | 0.808 | 0.820 |
|  | FGL | 0.714 | 0.609 | 0.660 | 0.578 | 0.817 | 0.677 | 0.747 | 0.665 |
|  | GL | 0.583 | 0.594 | 0.588 | 0.590 | 0.726 | 0.702 | 0.713 | 0.714 |
|  |  |  |  |  |  |  |  |  |  |
| 0.20 | CFGL | 0.718 | 0.671 | 0.691 | 0.612 | 0.818 | 0.810 | 0.789 | 0.722 |
|  | CFGLO | 0.721 | 0.729 | 0.724 | 0.735 | 0.818 | 0.765 | 0.814 | 0.828 |
|  | FGL | 0.721 | 0.589 | 0.653 | 0.550 | 0.818 | 0.649 | 0.735 | 0.631 |
|  | GL | 0.583 | 0.594 | 0.588 | 0.590 | 0.726 | 0.702 | 0.713 | 0.714 |
