# Supplementary material for: Condition-adaptive fused graphical lasso (CFGL): An adaptive procedure for inferring condition-specific gene co-expression network
Source: PLoS Comput Biol. 2018 Sep 21;14(9):e1006436. doi: 10.1371/journal.pcbi.1006436 (PMC6173447; doi:10.1371/journal.pcbi.1006436)
Supplement: S2 Table — (DOCX) [file pcbi.1006436.s008.docx]

**Supplementary Table 2. Comparison of partial AUCs in the 3-condition simulation studies.**

CFGL, FGL and GL were compared under 4 simulation scenarios (S1-S4) with n=50. The analysis was run over a grid of $\lambda_{1}$ and $\lambda_{2}$. The minimum BIC was achieved at $\lambda_{2}$=0.15. The ROC curves were computed over $\lambda_{1}$ with $\lambda_{2}$=0.15. The table summarizes the partial AUCs in the FPR range of [0, 0.05].

| Method | S1 | S2 | S3 | S4 |
| --- | --- | --- | --- | --- |
| CFGL | 0.649 | 0.508 | 0.650 | 0.513 |
| FGL | 0.605 | 0.494 | 0.615 | 0.499 |
| GL | 0.597 | 0.474 | 0.593 | 0.472 |
