# Supplementary material for: Condition-adaptive fused graphical lasso (CFGL): An adaptive procedure for inferring condition-specific gene co-expression network
Source: PLoS Comput Biol. 2018 Sep 21;14(9):e1006436. doi: 10.1371/journal.pcbi.1006436 (PMC6173447; doi:10.1371/journal.pcbi.1006436)
Supplement: S3 Table — (DOCX) [file pcbi.1006436.s009.docx]

**Supplementary Table 3. Numbers of detected co-expression edges in the rat multi-tissue dataset.**

The optimal BIC was achieved at $\lambda_{1}=$0.0010 and $\lambda_{2}=$0.0008 for CFGL and FGL, and $\lambda_{1}=$0.0009 for GL. We investigated the effect of $\lambda_{2}$by repeating the analysis for CFGL and FGL at $\lambda_{2}=$0.0010 and 0.0012. For WGCNA, we used the top 1000 edges in the brain tissue network and the top 500 edges in the heart network to ensure consistency in the number of edges with that of the estimated networks from CFGL/FGL.

| Method | $\lambda_{1}$ | $\lambda_{2}$ | Brain specific | Heart specific | Common |
| --- | --- | --- | --- | --- | --- |
| CFGL | 0.0010 | 0.0008 | 815 | 203 | 356 |
| FGL |  |  | 611 | 29 | 354 |
| CFGL | 0.0010 | 0.0010 | 446 | 293 | 522 |
| FGL |  |  | 233 | 15 | 522 |
| CFGL | 0.0010 | 0.0012 | 280 | 188 | 617 |
| FGL |  |  | 57 | 7 | 623 |
| GL | 0.0009 | - | 883 | 605 | 3 |
| WGCNA | - | - | 995 | 495 | 5 |
