# Supplementary material for: Condition-adaptive fused graphical lasso (CFGL): An adaptive procedure for inferring condition-specific gene co-expression network
Source: PLoS Comput Biol. 2018 Sep 21;14(9):e1006436. doi: 10.1371/journal.pcbi.1006436 (PMC6173447; doi:10.1371/journal.pcbi.1006436)
Supplement: S4 Table — (DOCX) [file pcbi.1006436.s010.docx]

**Supplementary Table 4. Top 5 tissue-specific hubs identified by GL with rat expression data.**

| Tissue | Hubs | CFGL | | FGL | | GL | | WGCNA | |
| --- | --- | --- | --- | --- | --- | --- | --- | --- | --- |
|  |  | #edge | #edge  ranking | #edge | #edge  Ranking | #edge | #edge  Ranking | #edge | #edge  Ranking |
| Brain | Fh12 | 4 | - | 3 | - | 67 | 1 | 0 | - |
|  | Neurod1 | 15 | 28 | 13 | 29 | 60 | 2 | 2 | - |
|  | Camkv | 0 | - | 0 | - | 34 | 3 | 1 | - |
|  | Elavl3 | 0 | - | 0 | - | 30 | 4 | 0 | - |
|  | Xirp2 | 4 | - | 0 | - | 27 | 5 | 0 | - |
| Heart | Cacna2d3 | 0 | - | 0 | - | 41 | 1 | 3 | - |
|  | Ckm | 0 | - | 0 | - | 35 | 2 | 8 | 15 |
|  | Olfm1 | 0 | - | 0 | - | 32 | 3 | 0 | - |
|  | Rit2 | 0 | - | 0 | - | 31 | 4 | 1 | - |
|  | Camkk2 | 0 | - | 0 | - | 30 | 5 | 3 | - |

‘-‘ means the ranking of edge are larger than 50
