# Supplementary material for: Condition-adaptive fused graphical lasso (CFGL): An adaptive procedure for inferring condition-specific gene co-expression network
Source: PLoS Comput Biol. 2018 Sep 21;14(9):e1006436. doi: 10.1371/journal.pcbi.1006436 (PMC6173447; doi:10.1371/journal.pcbi.1006436)
Supplement: S5 Table — (DOCX) [file pcbi.1006436.s011.docx]

**Supplementary Table 5. Top 5 tissue-specific hubs identified using topology overlapping matrix (TOM) estimated from WGCNA with rat expression data.**

| Tissue | Hubs | CFGL | | FGL | | GL | | WGCNA | |
| --- | --- | --- | --- | --- | --- | --- | --- | --- | --- |
|  |  | #edge | #edge  ranking | #edge | #edge  Ranking | #edge | #edge  Ranking | #edge | #edge  Ranking |
| Brain | Chn1 | 19 | 16 | 15 | 20 | 0 | - | 67 | 1 |
|  | Olfm1 | 0 | - | 0 | - | 0 | - | 58 | 2 |
|  | Atp5a1 | 0 | - | 0 | - | 0 | - | 53 | 3 |
|  | Slc24a2 | 0 | - | 0 | - | 0 | - | 49 | 4 |
|  | Cacng2 | 6 | - | 6 | - | 2 | - | 48 | 5 |
| Heart | My13 | 0 | - | 6 | - | 0 | - | 17 | 1 |
|  | Cacna1b | 0 | - | 0 | - | 0 | - | 15 | 2 |
|  | Atp5b | 0 | - | 0 | - | 0 | - | 13 | 3 |
|  | Mb | 0 | - | 0 | - | 5 | - | 12 | 4 |
|  | Anxa1 | 2 | 41 | 0 | - | 0 | - | 12 | 4 |

‘-‘ means the ranking of edge are larger than 50
