# Supplementary material for: Condition-adaptive fused graphical lasso (CFGL): An adaptive procedure for inferring condition-specific gene co-expression network
Source: PLoS Comput Biol. 2018 Sep 21;14(9):e1006436. doi: 10.1371/journal.pcbi.1006436 (PMC6173447; doi:10.1371/journal.pcbi.1006436)
Supplement: S6 Table — (DOCX) [file pcbi.1006436.s012.docx]

**Supplementary Table 6. GO-enrichment analysis result using co-expression modules identified by the WGCNA analysis with rat expression data.**

| Tissue | Module ID (nodes number) | GO term enrichment | FDR |
| --- | --- | --- | --- |
| Brain | Module 1 (26) | ion transmembrane transporter activity | 2.452E-2 |
|  |  | T-tubule organization | 8.101E-3 |
|  | Module 2 (102) | transmembrane transporter activity | 1.577E-10 |
|  |  | chemical synaptic transmission | 8.356E-12 |
|  | Module 3 (100) | clathrin binding | 5.806E-4 |
|  |  | synaptic signaling | 3.776E-11 |
|  | Module 4 (80) | channel activity | 3.481E-15 |
|  |  | regulation of ion transport | 2.297E-11 |
|  | Module 5 (60) | SNARE binding | 9.253E-4 |
|  |  | neuron projection morphogenesis | 7.223E-6 |
|  | Module 6 (57) | calmodulin binding | 5.883E-4 |
|  |  | chemical synaptic transmission | 5.569E-16 |
|  | Module 7 (37) | substantia nigra development | 1.302E-3 |
|  |  | neuron part | 1.436E-9 |
| Heart | Module 1 (202) | gated channel activity | 6.125E-16 |
|  |  | synaptic signaling | 7.512E-24 |
|  | Module 2 (178) | transmembrane transporter activity | 5.916E-14 |
|  |  | chemical synaptic transmission | 1.588E-26 |
|  | Module 3 (89) | channel activity | 6.141E-5 |
|  |  | chemical synaptic transmission | 3.631E-14 |
