# Supplementary material for: Condition-adaptive fused graphical lasso (CFGL): An adaptive procedure for inferring condition-specific gene co-expression network
Source: PLoS Comput Biol. 2018 Sep 21;14(9):e1006436. doi: 10.1371/journal.pcbi.1006436 (PMC6173447; doi:10.1371/journal.pcbi.1006436)
Supplement: S7 Table — (DOCX) [file pcbi.1006436.s013.docx]

**Supplementary Table 7. Disease type specificity of the estimated co-expression edges for TCGA breast cancer data.**

The networks from CFGL, FGL and GL were determined by performing each of the methods with a stability selection procedure. The edges from WGCNA were determined according to the TOM matrix estimated from WGCNA. Because the TOM matrix is not sparse, the edges with the top-n TOM values were selected for each tissue, where n is the number of edges in the CFGL networks.

|  | Normal only | ER+ tumor only | ER- tumor only | Shared in normal and ER+ | Shared in normal and ER- | Shared in ER+ and ER- | Shared in all tissue | Total number of edges |
| --- | --- | --- | --- | --- | --- | --- | --- | --- |
| CFGL | 384 | 554 | 360 | 250 | 60 | 332 | 684 | 2624 |
| FGL | 1034 | 840 | 472 | 446 | 100 | 226 | 1330 | 4448 |
| GL | 2250 | 2136 | 980 | 290 | 36 | 218 | 68 | 5978 |
| WGCNA | 874 | 1116 | 776 | 4 | 30 | 22 | 12 | 2834 |
