# Supplementary material for: Condition-adaptive fused graphical lasso (CFGL): An adaptive procedure for inferring condition-specific gene co-expression network
Source: PLoS Comput Biol. 2018 Sep 21;14(9):e1006436. doi: 10.1371/journal.pcbi.1006436 (PMC6173447; doi:10.1371/journal.pcbi.1006436)
Supplement: S8 Table — (DOCX) [file pcbi.1006436.s014.docx]

**Supplementary Table 8. Top hubs in ER+/ER-/tumor-shared network constructed using WGCNA with TCGA breast cancer data.**

Top 5 hubs that have the largest number of edges in each network. For tumor-shared network, there is only one gene (CCNT1) with more than three edges. The genes with three or fewer edges are not reported here.

| Tissue | Hubs (number of edges) |
| --- | --- |
| ER+/ER- shared | CCNT1(8) |
| ER+ specific | C1S (62), DZIP1(61), C1R(57), RECK(53), VIM (48) |
| ER- specific | CCNT1(99), SBNO1(63), RNF168(59), TAF1L(36), EXOC6B(29) |
