# Supplementary material for: Condition-adaptive fused graphical lasso (CFGL): An adaptive procedure for inferring condition-specific gene co-expression network
Source: PLoS Comput Biol. 2018 Sep 21;14(9):e1006436. doi: 10.1371/journal.pcbi.1006436 (PMC6173447; doi:10.1371/journal.pcbi.1006436)
Supplement: S9 Table — (DOCX) [file pcbi.1006436.s015.docx]

**Supplementary Table 9. GO-enrichment analysis results based on the co-expression modules identified by WGCNA in TCGA BRAC gene expression data.**

| Tissue | Module ID (nodes number) | GO term enrichment | FDR |
| --- | --- | --- | --- |
| Normal | Module 1 (351) | positive regulation of nucleobase-containing compound metabolic process | 2.443E-4 |
|  | Module 2 (325) | gland morphogenesis | 2.406E-4 |
|  |  | morphogenesis of a branching structure | 6.972E-3 |
|  | Module 3 (126) | adenyl nucleotide binding | 2.807E-4 |
|  |  | adenyl ribonucleotide binding | 3.257E-4 |
|  | Module 4 (88) | regulation of protein phosphorylation | 3.539E-2 |
| ER+ | Module 1 (557) | interspecies interaction between organisms | 4.993E-7 |
|  |  | symbiont process | 4.993E-7 |
|  | Module 2 (246) | regulation of programmed cell death | 3.464E-4 |
|  |  | biological adhesion | 3.464E-4 |
|  | Module 3 (79) | regulation of DNA recombination at telomere | 1.781E-2 |
| ER- | Module 1 (618) | DNA biosynthetic process | 1.937E-5 |
|  |  | chromosome organization | 1.937E-5 |
|  | Module 2 (152) | immune response | 6.021E-7 |
|  | Module 3 (126) | hexose metabolic process | 2.307E-3 |
|  |  | insulin receptor binding | 3.523E-2 |
