# Supplementary material for: Condition-adaptive fused graphical lasso (CFGL): An adaptive procedure for inferring condition-specific gene co-expression network
Source: PLoS Comput Biol. 2018 Sep 21;14(9):e1006436. doi: 10.1371/journal.pcbi.1006436 (PMC6173447; doi:10.1371/journal.pcbi.1006436)
Supplement: S10 Table — (DOCX) [file pcbi.1006436.s016.docx]

**Supplementary Table 10. List of the 39 genes that are known to be related to breast cancer and are included in our TCGA analysis**

| Gene | Source |  | Gene | Source |  | Gene | Source |
| --- | --- | --- | --- | --- | --- | --- | --- |
| FOXA1 | 1 |  | CDKN1B | 2 |  | BRCA2 | 2 |
| KIF2C | 1 |  | MAP2K4 | 2 |  | BRIP1 | 2 |
| AURKB | 1 |  | TBX3 | 2 |  | CHEK2 | 2 |
| RAD54L | 1 |  | CBFB | 2 |  | NBN | 2 |
| BUB1 | 1 |  | AFF2 | 2 |  | AKT1 | 2 |
| PTEN | 2 |  | PIK3R1 | 2 |  | AKT2 | 2 |
| AKT1 | 2 |  | PTPN22 | 2 |  | ESR1 | 2 |
| TP53 | 2 |  | PTPRD | 2 |  | MDM2 | 2 |
| GATA3 | 2 |  | NF1 | 2 |  | TUBB1 | 2 |
| CDH1 | 2 |  | SF3B1 | 2 |  | JAK2 | 2 |
| RB1 | 2 |  | CCND3 | 2 |  | EGFR | 2 |
| MLL3 | 2 |  | ATM | 2 |  | MAP2K1 | 2 |
| MAP3K1 | 2 |  | BRCA1 | 2 |  | CHEK1 | 2 |

Source 1: Peng, J., Wang, P., Zhou, N., & Zhu, J. (2009). Partial correlation estimation by joint sparse regression models. Journal of the American Statistical Association, 104(486), 735-746.

Source 2: Cancer Genome Atlas Network. (2012). Comprehensive molecular portraits of human breast tumors. Nature, 490(7418), 61
