# Supplementary material for: Condition-adaptive fused graphical lasso (CFGL): An adaptive procedure for inferring condition-specific gene co-expression network
Source: PLoS Comput Biol. 2018 Sep 21;14(9):e1006436. doi: 10.1371/journal.pcbi.1006436 (PMC6173447; doi:10.1371/journal.pcbi.1006436)
Supplement: S1 Text — (PDF) [file pcbi.1006436.s017.pdf]

# Appendix: ADMM Algorithm For CFGL

In this section, we present the details of algorithm to solve the problem (2)

$$\max_{\{\boldsymbol{\Theta}\}} \left( \sum_{k=1}^{K} n_k \left[ \log \left( \det(\boldsymbol{\Theta}^{(k)}) - \operatorname{tr}(\boldsymbol{S}^{(k)}\boldsymbol{\Theta}^{(k)}) \right) \right] - P\left\{\boldsymbol{\Theta}\right\} \right),$$

where the penalty term is the fused lasso

$$P\left\{\boldsymbol{\Theta}\right\} = \lambda_1 \sum_{k=1}^{K} \sum_{i \neq j} |\theta_{ij}^{(k)}| + \lambda_2 \sum_{k<k'}^{K} \sum_{i \neq j} w_{ij}^{(kk')} |\theta_{ij}^{(k)} - \theta_{ij}^{(k')}|.$$

Following [6], we solve problem (2) by using ADMM algorithm [69]. To proceed, we rewrite the problem as

$$\min_{\{\boldsymbol{\Theta}\},\{Z\}} \left( -\sum_{k=1}^{K} n_k \left[ \log \left( \det(\boldsymbol{\Theta}^{(k)}) - \operatorname{tr}\left( \boldsymbol{S}^{(k)}\boldsymbol{\Theta}^{(k)} \right) \right) \right] + P\{\boldsymbol{Z}\} \right)$$

subject to the positive definiteness constraint and $\boldsymbol{Z}^{(k)} = \boldsymbol{\Theta}^{k}$ for $k = 1, \ldots, K$, where $\{\boldsymbol{Z}\} = \left(\boldsymbol{Z}^{(1)}, \ldots, \boldsymbol{Z}^{(K)}\right)$. By [69], the scaled augmented Lagrangian for this problem is

$$\begin{aligned} L\left(\{\boldsymbol{\Theta}\}, \{\boldsymbol{Z}\}, \{\boldsymbol{U}\}\right) = & -\sum_{k=1}^{K} n_k \left[ \log \left( \det(\boldsymbol{\Theta}^{(k)}) - \operatorname{tr}\left( \boldsymbol{S}^{(k)}\boldsymbol{\Theta}^{(k)} \right) \right) \right] + P\{\boldsymbol{Z}\} \\ & + \frac{\rho}{2} \sum_{k=1}^{K} \|\boldsymbol{\Theta}^{(k)} - \boldsymbol{Z}^{(k)} + \boldsymbol{U}^{(k)}\|_F^2 - \frac{\rho}{2} \sum_{k=1}^{K} \|\boldsymbol{U}^{(k)}\|_F^2, \end{aligned}$$

where $\{\boldsymbol{U}\} = \boldsymbol{U}^{(1)}, \ldots, \boldsymbol{U}^{(k)}$ are dual variables and $\rho$ serves as a 'penalty parameter', and $\|\boldsymbol{\Theta}\|$ is the Frobenius norm of matrix $\boldsymbol{\Theta}$, $\|\boldsymbol{\Theta}\|_F^2 = \sum_i \sum_j \Theta_{ij}^2$.

Following the procedure of ADMM algorithm for JGL problem [6], we present the ADMM algorithm for solving problem (2).

Step 1 Initialize values: $\boldsymbol{\Theta}^{(k)} = \boldsymbol{I}, \boldsymbol{U}^{(k)} = \boldsymbol{0}$ and $\boldsymbol{Z}^{(k)} = \boldsymbol{0}$ for $k = 1, \ldots, K$. Set $\rho > 0$.

Step 2 For the $i$th step, update the following procedures.

(i) For $k = 1, \ldots, K$, update $\boldsymbol{\Theta}_{(i)}^{(k)}$ as the minimizete of

$$-n_k \left[\log(\det(\boldsymbol{\Theta}^{(k)})) - \mathrm{tr}(\boldsymbol{S}^{(k)}\boldsymbol{\Theta}^{(k)})\right] + \frac{\rho}{2}\|\boldsymbol{\Theta}^{(k)} - \boldsymbol{Z}_{(i-1)}^{(k)} + \boldsymbol{U}_{(i-1)}^{(k)}\|_F^2.$$

Let $\boldsymbol{V}\boldsymbol{D}\boldsymbol{V}^\top$ be the eigendecomposition of $\boldsymbol{S}^{(k)} - \rho\boldsymbol{Z}_{(i-1)}^{k}/n_k + \rho\boldsymbol{U}_{(i-1)}^{(k)}/n_k$, the solution is given by (Witten and Tibshirani, 2009) by $\boldsymbol{V}\widetilde{\boldsymbol{D}}\boldsymbol{V}^\top$, where $\widetilde{\boldsymbol{D}}$ is the diagonal matrix with $j$th diagonal element $\frac{n_k}{2\rho}\left[-D_{jj} + \sqrt{D_{jj}^2 + 4\rho/n_k}\right]$.

(ii) Update $\{\boldsymbol{Z}_{(i)}\}$ as the minimizer of

$$\frac{\rho}{2}\sum_{k=1}^{K}\|\boldsymbol{Z}^{(k)} - (\boldsymbol{\Theta}_{(i)}^{(k)} + \boldsymbol{U}_{(i-1)}^{(k)})\|_F^2 + P(\{\boldsymbol{Z}\}). \tag{S.1}$$

(iii) For $k = 1, \ldots, K$, update $\boldsymbol{U}_{(i)}^{(k)} = \boldsymbol{U}_{(i-1)}^{(k)} + \boldsymbol{\Theta}_{(i)}^{(k)} - \boldsymbol{Z}_{(i)}^{(k)}$.

Step 3 Repeat Step 2 until convergence.

*Remarks.*

(1) This algorithm is guaranteed to converge to the global optimum, by [69]. The positive definiteness constraint on the estimated precision matrices is naturally enforced by Step 2 (i).

(2) We use $\rho = 1$ for controlling the step size and declare convergence when

$$\sum_k \|\boldsymbol{\Theta}_{(i)}^{(k)} - \boldsymbol{\Theta}_{(i-1)}^{(k-1)}\|_1 / \sum_k \|\boldsymbol{\Theta}_{(i-1)}^{(k-1)}\|_1 < 10^{-5}.$$

(3) It remains the task of minimizing (S.1). Note that the problem (S.1) can be rewritten as

$$\min_{\{Z\}} \left[\frac{\rho}{2}\sum_{k=1}^{K}\|\boldsymbol{Z}^{(k)} - \boldsymbol{A}^{(k)}\|_F^2 + \lambda_1\sum_{k=1}^{K}\sum_{i\neq j}|Z_{ij}^{(k)}| + \lambda_2\sum_{k<k'}\sum_{i\neq j} w_{ij}^{(kk')}|Z_{ij}^{(k)} - Z_{ij}^{(k')}|\right], \tag{S.2}$$

where $\boldsymbol{A}^{(k)} = \boldsymbol{\Theta}^{(k)}_{(i)} + \boldsymbol{U}^{(k)}_{(i-1)}$.

Since the problem (S.2) is completely separable with respect to each pair of matrix element $(i,j)$, one can simply solve the problem

$$\min_{Z^{(1)}_{ij},\ldots,Z^{(K)}_{ij}} \left[ \frac{\rho}{2} \sum_{k=1}^{K} (\boldsymbol{Z}^{(k)}_{ij} - \boldsymbol{A}^{(k)}_{ij}\|_F^2 + \lambda_1 \sum_{k=1}^{K} 1_{i\neq j}|Z^{(k)}_{ij}| + \lambda_2 \sum_{k<k'} w^{(kk')}_{ij} |Z^{(k)}_{ij} - Z^{(k')}_{ij}| \right], \qquad \text{(S.3)}$$

for each $(i,j)$.

Let $\widehat{\boldsymbol{Z}}(\lambda_1,\lambda_2) = \left(\widehat{Z}^{(1)}_{ij}(\lambda_1,\lambda_2),\ldots,\widehat{Z}^{(K)}_{ij}(\lambda_1,\lambda_2)\right)^\top$ be the solution to the problem (S.3). Note that $\widehat{\boldsymbol{Z}}(\lambda_1,\lambda_2)$ depends on $\lambda_1$ and $\lambda_2$. By [S1,S2], $\widehat{\boldsymbol{Z}}(\lambda_1,\lambda_2) = \mathrm{SH}\left(\widehat{\boldsymbol{Z}}(0,\lambda_2), \frac{\lambda_1}{\rho}\right)$, where the soft thresholding operator $\mathrm{SH}(x,\lambda)$ is defined by

$$\mathrm{SH}(x,\lambda) = \begin{cases} x+\lambda, & x<\lambda, \\ 0, & -\lambda < x < \lambda, \\ x-\lambda, & x>\lambda. \end{cases}$$

We present two examples for illustration to derive $\widehat{\boldsymbol{Z}}(0,\lambda_2)$ for $K=2$ and $K=3$, respectively.

**Example 1**. For $K=2$, the solution $\widehat{\boldsymbol{Z}}(0,\lambda_2) = \left(\widehat{Z}^{(1)}_{ij}, \widehat{Z}^{(2)}_{ij}\right)$ takes the form

(1) If $A^{(1)}_{ij} - A^{(2)}_{ij} > \frac{2\lambda_2}{\rho} w^{(12)}_{ij}$, then

$$\left(\widehat{Z}^{(1)}_{ij}, \widehat{Z}^{(2)}_{ij}\right) = \left(A^{(1)}_{ij} - \frac{\lambda_2}{\rho} w^{(12)}_{ij}, A^{(1)}_{ij} + \frac{\lambda_2}{\rho} w^{(12)}_{ij}\right);$$

(2) If $A^{(1)}_{ij} - A^{(2)}_{ij} < -\frac{2\lambda_2}{\rho} w^{(12)}_{ij}$, then

$$\left(\widehat{Z}^{(1)}_{ij}, \widehat{Z}^{(2)}_{ij}\right) = \left(A^{(1)}_{ij} + \frac{\lambda_2}{\rho} w^{(12)}_{ij}, A^{(1)}_{ij} - \frac{\lambda_2}{\rho} w^{(12)}_{ij}\right);$$

(3) If $|A^{(1)}_{ij} - A^{(2)}_{ij}| \leq \frac{2\lambda_2}{\rho} w^{(12)}_{ij}$, then

$$\left(\widehat{Z}^{(1)}_{ij}, \widehat{Z}^{(2)}_{ij}\right) = \left(\frac{A^{(1)}_{ij} + A^{(2)}_{ij}}{2}, \frac{A^{(1)}_{ij} + A^{(2)}_{ij}}{2}\right).$$

**Example 2**. For $K=3$, the solution $\widehat{\boldsymbol{Z}}(0,\lambda_2) = \left(\widehat{Z}^{(1)}_{ij}, \widehat{Z}^{(2)}_{ij}, \widehat{Z}^{(3)}_{ij}\right)$ takes the form

(1) If $A_{ij}^{(1)} - \frac{\lambda_2}{\rho} w_{ij}^{(12)} - \frac{\lambda_2}{\rho} w_{ij}^{(13)} > A_{ij}^{(2)} + \frac{\lambda_2}{\rho} w_{ij}^{(12)} - \frac{\lambda_2}{\rho} w_{ij}^{(23)} > A_{ij}^{(3)} + \frac{\lambda_2}{\rho} w_{ij}^{(23)} + \frac{\lambda_2}{\rho} w_{ij}^{(13)}$, then

$$\left(\widehat{Z}_{ij}^{(1)}, \widehat{Z}_{ij}^{(2)}, \widehat{Z}_{ij}^{(3)}\right) = \left(A_{ij}^{(1)} - \frac{\lambda_2}{\rho} w_{ij}^{(12)} - \frac{\lambda_2}{\rho} w_{ij}^{(13)}, A_{ij}^{(2)} + \frac{\lambda_2}{\rho} w_{ij}^{(12)} - \frac{\lambda_2}{\rho} w_{ij}^{(23)}, A_{ij}^{(3)} + \frac{\lambda_2}{\rho} w_{ij}^{(23)} + \frac{\lambda_2}{\rho} w_{ij}^{(13)}\right);$$

(2) If $|A_{ij}^{(1)} - A_{ij}^{(2)} - \frac{\lambda_2}{\rho} w_{ij}^{(13)} + \frac{\lambda_2}{\rho} w_{ij}^{(23)}| \leq \frac{\lambda_2}{\rho} w_{ij}^{(12)}$, and

$$\frac{A_{ij}^{(1)} + A_{ij}^{(2)} - \frac{\lambda_2}{\rho}(w_{ij}^{(13)} + w_{ij}^{(23)})}{2} > A_{ij}^{(3)} + \frac{\lambda_2}{\rho}(w_{ij}^{(23)} + w_{ij}^{(13)}),$$

then

$$\begin{aligned} &\left(\widehat{Z}_{ij}^{(1)}, \widehat{Z}_{ij}^{(2)}, \widehat{Z}_{ij}^{(3)}\right) \\ = &\left(\frac{A_{ij}^{(1)} + A_{ij}^{(2)} - \frac{\lambda_2}{\rho}(w_{ij}^{(13)} + w_{ij}^{(23)})}{2}, \frac{A_{ij}^{(1)} + A_{ij}^{(2)} - \frac{\lambda_2}{\rho}(w_{ij}^{(13)} + w_{ij}^{(23)})}{2}, A_{ij}^{(3)} + \frac{\lambda_2}{\rho}(w_{ij}^{(23)} + w_{ij}^{(13)})\right); \end{aligned}$$

(3) If $|A_{ij}^{(2)} - A_{ij}^{(3)} + \frac{\lambda_2}{\rho} w_{ij}^{(12)} - \frac{\lambda_2}{\rho} w_{ij}^{(13)}| \leq \frac{\lambda_2}{\rho} w_{ij}^{(23)}$, and

$$\frac{A_{ij}^{(2)} + A_{ij}^{(3)} + \frac{\lambda_2}{\rho}(w_{ij}^{(12)} + w_{ij}^{(13)})}{2} < A_{ij}^{(1)} - \frac{\lambda_2}{\rho}(w_{ij}^{(12)} + w_{ij}^{(13)}),$$

then

$$\begin{aligned} &\left(\widehat{Z}_{ij}^{(1)}, \widehat{Z}_{ij}^{(2)}, \widehat{Z}_{ij}^{(3)}\right) \\ = &\left(A_{ij}^{(1)} - \frac{\lambda_2}{\rho}(w_{ij}^{(12)} + w_{ij}^{(13)}), \frac{A_{ij}^{(2)} + A_{ij}^{(3)} - \frac{\lambda_2}{\rho}(w_{ij}^{(12)} + w_{ij}^{(13)})}{2}, \frac{A_{ij}^{(2)} + A_{ij}^{(3)} - \frac{\lambda_2}{\rho}(w_{ij}^{(12)} + w_{ij}^{(13)})}{2}\right); \end{aligned}$$

(4) If $A_{ij}^{(1)} - \frac{\lambda_2}{\rho} w_{ij}^{(12)} - \frac{\lambda_2}{\rho} w_{ij}^{(13)} > A_{ij}^{(3)} - \frac{\lambda_2}{\rho} w_{ij}^{(23)} + \frac{\lambda_2}{\rho} w_{ij}^{(13)} > A_{ij}^{(2)} + \frac{\lambda_2}{\rho} w_{ij}^{(12)} + \frac{\lambda_2}{\rho} w_{ij}^{(23)}$, then

$$\left(\widehat{Z}_{ij}^{(1)}, \widehat{Z}_{ij}^{(2)}, \widehat{Z}_{ij}^{(3)}\right) = \left(A_{ij}^{(1)} - \frac{\lambda_2}{\rho} w_{ij}^{(12)} - \frac{\lambda_2}{\rho} w_{ij}^{(13)}, A_{ij}^{(2)} + \frac{\lambda_2}{\rho} w_{ij}^{(12)} + \frac{\lambda_2}{\rho} w_{ij}^{(23)}, A_{ij}^{(3)} - \frac{\lambda_2}{\rho} w_{ij}^{(23)} + \frac{\lambda_2}{\rho} w_{ij}^{(13)}\right);$$

(5) If $|A_{ij}^{(1)} - A_{ij}^{(3)} - \frac{\lambda_2}{\rho} w_{ij}^{(12)} + \frac{\lambda_2}{\rho} w_{ij}^{(23)}| \leq \frac{\lambda_2}{\rho} w_{ij}^{(13)}$, and

$$\frac{A_{ij}^{(1)} + A_{ij}^{(3)} - \frac{\lambda_2}{\rho}(w_{ij}^{(12)} + w_{ij}^{(23)})}{2} > A_{ij}^{(2)} + \frac{\lambda_2}{\rho}(w_{ij}^{(12)} + w_{ij}^{(23)}),$$

then

$$\left(\widehat{Z}_{ij}^{(1)}, \widehat{Z}_{ij}^{(2)}, \widehat{Z}_{ij}^{(3)}\right)$$
$$= \left(\frac{A_{ij}^{(1)} + A_{ij}^{(3)} - \frac{\lambda_2}{\rho}(w_{ij}^{(12)} + w_{ij}^{(23)})}{2}, A_{ij}^{(2)} + \frac{\lambda_2}{\rho}(w_{ij}^{(12)} + w_{ij}^{(23)}), \frac{A_{ij}^{(1)} + A_{ij}^{(3)} - \frac{\lambda_2}{\rho}(w_{ij}^{(12)} + w_{ij}^{(23)})}{2}\right);$$

(6) If $A_{ij}^{(2)} - \frac{\lambda_2}{\rho} w_{ij}^{(12)} - \frac{\lambda_2}{\rho} w_{ij}^{(23)} > A_{ij}^{(1)} + \frac{\lambda_2}{\rho} w_{ij}^{(12)} - \frac{\lambda_2}{\rho} w_{ij}^{(13)} > A_{ij}^{(3)} + \frac{\lambda_2}{\rho} w_{ij}^{(23)} + \frac{\lambda_2}{\rho} w_{ij}^{(13)}$, then

$$\left(\widehat{Z}_{ij}^{(1)}, \widehat{Z}_{ij}^{(2)}, \widehat{Z}_{ij}^{(3)}\right) = \left(A_{ij}^{(1)} + \frac{\lambda_2}{\rho} w_{ij}^{(12)} - \frac{\lambda_2}{\rho} w_{ij}^{(13)}, A_{ij}^{(2)} - \frac{\lambda_2}{\rho} w_{ij}^{(12)} - \frac{\lambda_2}{\rho} w_{ij}^{(23)}, A_{ij}^{(3)} + \frac{\lambda_2}{\rho} w_{ij}^{(23)} + \frac{\lambda_2}{\rho} w_{ij}^{(13)}\right);$$

(7) If $|A_{ij}^{(1)} - A_{ij}^{(3)} + \frac{\lambda_2}{\rho} w_{ij}^{(12)} - \frac{\lambda_2}{\rho} w_{ij}^{(23)}| \leq \frac{\lambda_2}{\rho} w_{ij}^{(13)}$, and

$$\frac{A_{ij}^{(1)} + A_{ij}^{(3)} + \frac{\lambda_2}{\rho}(w_{ij}^{(12)} + w_{ij}^{(23)})}{2} < A_{ij}^{(2)} - \frac{\lambda_2}{\rho}(w_{ij}^{(12)} + w_{ij}^{(23)}),$$

then

$$\left(\widehat{Z}_{ij}^{(1)}, \widehat{Z}_{ij}^{(2)}, \widehat{Z}_{ij}^{(3)}\right)$$
$$= \left(\frac{A_{ij}^{(1)} + A_{ij}^{(3)} + \frac{\lambda_2}{\rho}(w_{ij}^{(12)} + w_{ij}^{(23)})}{2}, A_{ij}^{(2)} - \frac{\lambda_2}{\rho}(w_{ij}^{(12)} + w_{ij}^{(23)}), \frac{A_{ij}^{(1)} + A_{ij}^{(3)} + \frac{\lambda_2}{\rho}(w_{ij}^{(12)} + w_{ij}^{(23)})}{2}\right);$$

(8) If $A_{ij}^{(2)} - \frac{\lambda_2}{\rho} w_{ij}^{(12)} - \frac{\lambda_2}{\rho} w_{ij}^{(23)} > A_{ij}^{(3)} + \frac{\lambda_2}{\rho} w_{ij}^{(23)} - \frac{\lambda_2}{\rho} w_{ij}^{(13)} > A_{ij}^{(1)} + \frac{\lambda_2}{\rho} w_{ij}^{(12)} + \frac{\lambda_2}{\rho} w_{ij}^{(13)}$, then

$$\left(\widehat{Z}_{ij}^{(1)}, \widehat{Z}_{ij}^{(2)}, \widehat{Z}_{ij}^{(3)}\right) = \left(A_{ij}^{(1)} + \frac{\lambda_2}{\rho} w_{ij}^{(12)} + \frac{\lambda_2}{\rho} w_{ij}^{(13)}, A_{ij}^{(2)} - \frac{\lambda_2}{\rho} w_{ij}^{(12)} - \frac{\lambda_2}{\rho} w_{ij}^{(23)}, A_{ij}^{(3)} + \frac{\lambda_2}{\rho} w_{ij}^{(23)} - \frac{\lambda_2}{\rho} w_{ij}^{(13)}\right);$$

(9) If $|A_{ij}^{(2)} - A_{ij}^{(3)} - \frac{\lambda_2}{\rho} w_{ij}^{(12)} + \frac{\lambda_2}{\rho} w_{ij}^{(13)}| \leq \frac{\lambda_2}{\rho} w_{ij}^{(23)}$, and

$$\frac{A_{ij}^{(2)} + A_{ij}^{(3)} - \frac{\lambda_2}{\rho}(w_{ij}^{(12)} + w_{ij}^{(13)})}{2} > A_{ij}^{(1)} + \frac{\lambda_2}{\rho}(w_{ij}^{(12)} + w_{ij}^{(13)}),$$

then

$$\begin{aligned}&\left(\widehat{Z}_{ij}^{(1)},\widehat{Z}_{ij}^{(2)},\widehat{Z}_{ij}^{(3)}\right)\\ =&\left(A_{ij}^{(1)}+\frac{\lambda_2}{\rho}(w_{ij}^{(12)}+w_{ij}^{(13)}),\frac{A_{ij}^{(2)}+A_{ij}^{(3)}-\frac{\lambda_2}{\rho}(w_{ij}^{(12)}+w_{ij}^{(13)})}{2},\frac{A_{ij}^{(2)}+A_{ij}^{(3)}-\frac{\lambda_2}{\rho}(w_{ij}^{(12)}+w_{ij}^{(13)})}{2}\right);\end{aligned}$$

(10) If $A_{ij}^{(3)}-\frac{\lambda_2}{\rho}w_{ij}^{(23)}-\frac{\lambda_2}{\rho}w_{ij}^{(13)}>A_{ij}^{(1)}-\frac{\lambda_2}{\rho}w_{ij}^{(12)}+\frac{\lambda_2}{\rho}w_{ij}^{(13)}>A_{ij}^{(2)}+\frac{\lambda_2}{\rho}w_{ij}^{(12)}+\frac{\lambda_2}{\rho}w_{ij}^{(23)}$, then

$$\left(\widehat{Z}_{ij}^{(1)},\widehat{Z}_{ij}^{(2)},\widehat{Z}_{ij}^{(3)}\right)=\left(A_{ij}^{(1)}-\frac{\lambda_2}{\rho}w_{ij}^{(12)}+\frac{\lambda_2}{\rho}w_{ij}^{(13)},A_{ij}^{(2)}+\frac{\lambda_2}{\rho}w_{ij}^{(12)}+\frac{\lambda_2}{\rho}w_{ij}^{(23)},A_{ij}^{(3)}-\frac{\lambda_2}{\rho}w_{ij}^{(23)}-\frac{\lambda_2}{\rho}w_{ij}^{(13)}\right);$$

(11) If $|A_{ij}^{(1)}-A_{ij}^{(2)}+\frac{\lambda_2}{\rho}w_{ij}^{(13)}-\frac{\lambda_2}{\rho}w_{ij}^{(23)}|\leq\frac{\lambda_2}{\rho}w_{ij}^{(12)}$, and

$$\frac{A_{ij}^{(1)}+A_{ij}^{(2)}+\frac{\lambda_2}{\rho}(w_{ij}^{(13)}+w_{ij}^{(23)})}{2}<A_{ij}^{(3)}-\frac{\lambda_2}{\rho}(w_{ij}^{(23)}-w_{ij}^{(13)}),$$

then

$$\begin{aligned}&\left(\widehat{Z}_{ij}^{(1)},\widehat{Z}_{ij}^{(2)},\widehat{Z}_{ij}^{(3)}\right)\\ =&\left(\frac{A_{ij}^{(1)}+A_{ij}^{(2)}+\frac{\lambda_2}{\rho}(w_{ij}^{(13)}+w_{ij}^{(23)})}{2},\frac{A_{ij}^{(1)}+A_{ij}^{(2)}+\frac{\lambda_2}{\rho}(w_{ij}^{(13)}+w_{ij}^{(23)})}{2},A_{ij}^{(3)}-\frac{\lambda_2}{\rho}(w_{ij}^{(23)}-w_{ij}^{(13)})\right);\end{aligned}$$

(12) If $A_{ij}^{(3)}-\frac{\lambda_2}{\rho}w_{ij}^{(23)}-\frac{\lambda_2}{\rho}w_{ij}^{(13)}>A_{ij}^{(2)}-\frac{\lambda_2}{\rho}w_{ij}^{(12)}+\frac{\lambda_2}{\rho}w_{ij}^{(23)}>A_{ij}^{(1)}+\frac{\lambda_2}{\rho}w_{ij}^{(12)}+\frac{\lambda_2}{\rho}w_{ij}^{(13)}$, then

$$\left(\widehat{Z}_{ij}^{(1)},\widehat{Z}_{ij}^{(2)},\widehat{Z}_{ij}^{(3)}\right)=\left(A_{ij}^{(1)}+\frac{\lambda_2}{\rho}w_{ij}^{(12)}+\frac{\lambda_2}{\rho}w_{ij}^{(13)},A_{ij}^{(2)}-\frac{\lambda_2}{\rho}w_{ij}^{(12)}+\frac{\lambda_2}{\rho}w_{ij}^{(23)},A_{ij}^{(3)}-\frac{\lambda_2}{\rho}w_{ij}^{(23)}-\frac{\lambda_2}{\rho}w_{ij}^{(13)}\right);$$

(13) If

$$\begin{aligned}|A_{ij}^{(1)}+A_{ij}^{(2)}-2A_{ij}^{(3)}| &\leq \frac{3\lambda_2}{\rho}(w_{ij}^{(13)}+w_{ij}^{(23)}),\\ |A_{ij}^{(1)}+A_{ij}^{(3)}-2A_{ij}^{(2)}| &\leq \frac{3\lambda_2}{\rho}(w_{ij}^{(12)}+w_{ij}^{(23)}),\\ |A_{ij}^{(2)}+A_{ij}^{(3)}-2A_{ij}^{(1)}| &\leq \frac{3\lambda_2}{\rho}(w_{ij}^{(12)}+w_{ij}^{(13)}),\end{aligned}$$

then

$$\left(\widehat{Z}_{ij}^{(1)}, \widehat{Z}_{ij}^{(2)}, \widehat{Z}_{ij}^{(3)}\right) = \left(\frac{A_{ij}^{(1)} + A_{ij}^{(2)} + A_{ij}^{(3)}}{3}, \frac{A_{ij}^{(1)} + A_{ij}^{(2)} + A_{ij}^{(3)}}{3}, \frac{A_{ij}^{(1)} + A_{ij}^{(2)} + A_{ij}^{(3)}}{3}\right).$$

# References


S1. Friedman, J., Hastie, T., Hoefling, H. and Tibshirani, R. (2007). Pathwise coordinate optimization. *The Annals of Applied Statistics*, 1, 302–332.

S2. Tibshirani, R., Saunders, M., Rosset, S., Zhu, J. and Knight, K. (2005). Sparsity and smoothness via the fused lasso. *Journal of the Royal Statistical Society, Series B*, 67, 91–108.
